# Radiographic Identification of Osseous Cyst- Like Lesions in the Distal Phalanx in 22 Lame Thoroughbred Horses Managed Conservatively and Their Racing Performance

**DOI:** 10.3389/fvets.2018.00286

**Published:** 2018-11-22

**Authors:** Vanessa G. Peter, Thomas A. O'Keeffe, Lewis C. R. Smith, Daniela Schweizer-Gorgas

**Affiliations:** ^1^Division of Clinical Radiology, Department of Clinical Veterinary Science, Vetsuisse Faculty, University of Bern, Bern, Switzerland; ^2^Rossdales Equine Hospital and Diagnostic Centre, Newmarket, United Kingdom

**Keywords:** bone cyst, pedal, radiography, equine, racehorse

## Abstract

**Reasons for performing study:** To investigate the racing performance of Thoroughbred horses with osseous cyst-like lesions (OCLLs) in the distal phalanx causing lameness and treated conservatively.

**Objectives:** To assess horses' ability to race and perform after radiographic identification of OCLL in the distal phalanx of Thoroughbred horses with lameness at the time of detection and undergoing conservative treatment.

**Study Design:** Retrospective case control study.

**Methods:** The clinical database of one equine clinic was reviewed in a 10-year period for Thoroughbreds showing lameness localized to the foot and a radiographic diagnosis of OCLL in the distal phalanx. Sex, age at time of detection of the OCLL, degree of lameness, affected limb, and treatment were recorded. Successful performance of horses was assessed by racing at least once after detection of the OCLL and maximum racing performance rating (RPR). Radiographic features such as size, location, sclerotic rim of the OCLL and irregularity of the articular surface of the distal phalanx were compared to successful performance using univariable statistical analysis. Successful performance of horses with OCLL was compared to a control group of maternal siblings by parametric testing.

**Results:** Twenty-two horses met the inclusion criteria. Thirteen horses raced after the detection of OCLLs. Eight did not race, one case had not yet reached racing age, resulting in 62% (13/21) of racing age racing at least once. The number of successfully performing horses with an OCCL was significantly lower compared to their maternal siblings [*p* = 0.03, Odds ratio (OR) = 0.30]. If horses with OCLL in the distal phalanx raced, their RPR was similar to their maternal siblings. No significant association was found between radiographic features of OCLLs and successful performance, but OCLLs in the left forelimb carried a more favorable outcome for racing (*p* = 0.02, OR = 2.33 95%CI 1.27, 4.27) compared to OCLLs in any other limb.

**Conclusions:** Horses with lameness and an OCLL in the distal phalanx managed conservatively are less likely to race when compared to their maternal siblings. If horses with OCLLs in the distal phalanx are able to race, their performance, measured as RPR, was comparable to their maternal siblings. Due to the small numbers in this study the results should be interpreted carefully.

## Introduction

Lameness in racehorses results in welfare implications for the affected individual as well as in the loss of training days leading to poor performance and financial loss for owners. Lameness is therefore a major cause of wastage in the Thoroughbred racehorse (1). In lame Thoroughbred racehorses, the veterinarian is expected to establish a diagnosis, which allows them to provide an accurate prognosis on racing performance, whilst choosing a cost effective, practical diagnostic tool, appropriate for use in an ambulatory setting. One possible cause of lameness of the distal limb is an OCLL within the distal phalanx (2, 3).

OCLLs are described at various locations such as in the middle and proximal phalanx, the carpal bones, the third metacarpal/metatarsal bones, the tibia, radius, talus, sesamoid bones, humerus, patella, scapula, tarsal bones, femoral head, and hemimandible (4–12), but the most common location in horses is the medial femoral condyle, followed by the distal phalanx (8). Within the distal phalanx, OCLLs may develop at various locations but are most commonly located adjacent to the articular surface, with or without a communication to the joint and often found at the center of the proximal aspect of the distal phalanx. Since they lack an epithelial lining, they are not considered as true bone cysts (13, 14). The pathogenesis of such OCLLs is not completely understood, but a developmental disorder with failure of endochondral ossification, trauma, focal sepsis and/or ischemia, are considered as potential underlying pathomechanisms (15, 16).

After localization of a lameness, radiography is usually the first imaging modality used as it is cost effective and readily available. OCLLs may be detected on standard radiographic views (3, 11). Dependent on their size and location, OCLLs may be visible as a small radiolucent flattening or depression in the articular surface or can develop to circular, oval or conical radiolucencies, possibly surrounded by a sclerotic rim (3, 11). Treatment recommendations for OCLLs often depend on personal preference as well as financial considerations. In cases where the OCLL is suspected to communicate with the joint space, intra articular injection with corticosteroids is commonly used as a first line treatment (2, 17). However, treatment response to conservative treatment and return to athletic function has been investigated on limited numbers of horses with different usage, and variable outcomes are reported when the OCLL was found in the distal phalanx or the stifle (13). Treatment options, such as the ultrasound-guided injection of corticosteroids into the cyst lining are not applicable to OCLLs in the distal phalanx because of the surrounding hoof capsule. Arthroscopic debridement of the OCLL in the distal phalanx of 11 horses, where conservative treatment failed, resulted in 91% returning to athletic performance (17). The population of this study included nine Thoroughbreds. Eight went on to be athletic and seven raced successfully, with earning of the horses not statistically different from the maternal siblings (17). All publications of OCLLs in the distal phalanx are based on rather small numbers, and the racing performance has only been evaluated after surgical treatment (13, 17–20). Since there is a large Thoroughbred horse population in the UK, the aim of the present study was to assess the racing performance of Thoroughbred horses presented for lameness localized to the foot and with a radiographic diagnosis of an OCLL managed with rest or medical treatment alone. Our first hypothesis was that lame Thoroughbred racehorses with OCLLs in the distal phalanx managed conservatively would be less likely to race then their maternal siblings. Our second hypothesis was that the affected horses would perform less successful than their maternal siblings.

## Materials and methods

The clinical database of a specialized equine clinic (Rossdales LLP, Newmarket, UK) was searched from 2008 to 2018 for Thoroughbred horses presented with fore- or hindlimb lameness and the radiographic diagnosis of an OCLL affecting the distal phalanx. A further inclusion criteria was a clinical lameness examination which localized the lameness to the foot with an intra-articular anesthesia of the distal interphalangeal joint, a positive palmar/plantar digital nerve block or an abaxial sesamoid nerve block with a negative response to metacarpo-/metatarsophalangeal joint anesthesia, as well as conservative treatment of the lameness. Sex, age at time of detection of the cyst, degree of lameness (AAEP grading scale), affected limb, and choice of treatment were recorded. Treatment options were intra-articular injection of triamcinolone and hyaluronic acid as a combined injection, triamcinolone alone or platelet rich plasma.

Radiographs of the foot were obtained with either a computed radiographic system (Agfa DX-G, Agfa HealthCare, Belgium) or a direct digital radiographic system (Tru-DR Sound, Canon Imaging Systems, Japan). Radiographs of the distal limb of the included horses were reviewed in DICOM format using Horos software [v 2.2.0 (2017), Horos Project, USA] on a medical grade imaging monitor (EIZO, Japan). Radiographs were evaluated by a third year resident in diagnostic imaging (VP) and a board-certified radiologist (DSG) for the number, size (in mm) and location of the OCLL within the distal phalanx (sagittal, lateral or medial), presence of a sclerotic rim of the OCLL (yes/no) and presence of an irregular articular surface of the distal phalanx in proximity to the OCLL (yes/no). It was noted if the lesion was visible on the lateromedial or oblique projections as an indentation of the articular surface of the distal phalanx and if this indentation was found to be located in the dorsal, middle or palmar/plantar aspect of the joint. Radiographs were also evaluated for changes in the adjacent structures such as the presence of enthesophyte formation at the dorsodistal aspect of the middle phalanx, osteophyte formation affecting the distal interphalangeal joint and increased soft tissue opacity dorsal to the distal interphalangeal joint.

Successful performance of the horses was assessed by racing at least once after detection of the cyst (yes/no) and RPR. The RPR is one measure to assess racing performance in the Thoroughbred in the UK (21–24). The RPR is published by the *Racing Post* newspaper and is calculated after every race taking the distance won/beaten in lengths, the collateral form (performance and rating of other horses participating in the race) and the weight the horse carried into account. The RPR does not reflect a horse‘s potential, it reflects the horse's maximum effort in a race (24). The RPR is correlated with racing earning (*P* < 0.001) (21). We used the highest RPR of each horse published as a measurement of the best performance. The control group consisted of all maternal siblings of the affected horses, identified by the online database of the racing post. Affected and control horses' race histories and their highest RPR were recorded.

Combined regression analysis was performed in the group of maternal siblings to allow for clustering of data by dam. Horses' age at detection of lameness, sex, affected limb, forelimb vs. hindlimb affected, grade of lameness, and successful racing performance, as well as radiographic features of the OCLL, were assessed for potential associations with the binary outcome of racing or not racing as the dependent variable. If horses raced at least once, their RPR was compared to their maternal siblings. The continuous variables (age and RPR) were analyzed for normality using a Shapiro-Wilk test and underwent parametric testing with an unpaired *T*-test to assess for statistical significance. Univariable statistical analysis of categorical data using a Chi-squared or Fisher's exact test was performed as appropriate to identify statistically significant variables. The level of significance was set to *p* < 0.05. Odds ratios (OR) with 95% confidence intervals (CI) were calculated. *Post hoc* power analysis was performed on horses of racing age for the dichotomous endpoint of racing or not racing in the group of cases (*n* = 21) and the maternal siblings group (*n* = 141) as well as for the continuous endpoint of the RPR mean for the 13 cases that raced, with a standard deviation of 23.6 and the 53 maternal siblings in the control group with a standard deviation of 28.3. The type I/II error Rate was defined as 0.05 (alpha = 0.05). Possible statistical significance of the choice of treatment, influence of dam on the racing performance of maternal siblings and the outcome of raced or not raced was assessed by linear regression analysis.

## Results

Of the 20,056 Thoroughbred horses presented because of lameness at the specialized equine clinic within 10 years, 22 cases met the inclusion criteria: 11 male and 11 female horses. These included two foals, eight yearlings, nine two-year-old horses, one three-year-old and two horses being at least 4-years-old with a median age of 2 years and a mean age of 2.1 years (summarized in Table 1). The OCLLs were identified in the forelimb in 17 horses and in the hindlimb in five horses. In two horses, an OCLL was identified in the contralateral non-lame limb, both were affecting hindlimbs.

**Table 1:** Case list of the 22 Thoroughbred horses with radiographically evident OCLL: Age at time of detection, sex, lameness grade, affected limb, diagnostic anesthesia performed, irregular articular margin, treatment and outcome.

**Case No**	**Age in years**	**Sex**	**AAEP lameness grade**	**Lame limb**	**Positive diagnostic anesthesia**	**OCLL location in the distal phalanx**	**Irregular articular margin on radiographs**	**Treatment**	**Raced after detection of the OCLL**
1	1	Female	3	RF	DIPJ	Lateral	No	Steroids	No
2	1	Female	3	LF	DIPJ	Sagittal	No	PRP	Yes
3	1	Male	4	RF	PDNB	Sagittal	Yes	Steroids	Yes
4	0.5	Female	3	RH	ASNB	Sagittal	Yes	PRP	N/A
5	5	Female	2	RF	DIPJ	Medial	No	Steroids	Yes
6	9	Male	3	LF	PDNB	Sagittal	No	HA and steroids	Yes
7	1	Male	2	LF	PDNB	Sagittal	No	HA and steroids	Yes
8	3	Male	4	RF	PDNB	Medial	No	HA and steroids	Yes
9	1	Male	3	RH	ASNB	Sagittal	No	HA and steroids	No
10	2	Male	2	LF	DIPJ	Sagittal	Yes	HA and steroids	Yes
11	2	Male	2	LF	DIPJ	Medial	No	HA and steroids	Yes
12	1	Female	3	LF	DIPJ	Sagittal	Yes	HA and steroids	Yes
13	2	Female	3	RH	ASNB	Medial	No	HA and steroids	No
14	1	Female	3	RF	DIPJ	Sagittal	No	PRP	Yes
15	2	Female	2	RF	DIPJ	Sagittal	No	PRP	Yes
16	0.5	Male	3	RH	ASNB	Sagittal	Yes	Steroids	No
17	2	Male	3	RF	DIPJ	Sagittal	Yes	Rest only	No
18	1	Female	3	RF	DIPJ	Sagittal	No	HA and steroids	No
19	2	Male	3	RF	DIPJ	Lateral	No	Rest only	No
20	12	Female	2	LF	DIPJ	Sagittal	Yes	HA and steroids	Yes
21	2	Female	3	RF	DIPJ	Sagittal	No	HA and steroids	No
22	2	Male	1	LH	PDNB	Sagittal	Yes	HA and steroids	Yes

*LF, left forelimb; RF, right forelimb; LH, left hindlimb; RH, right hindlimb; DIPJ, distal interphalangeal joint; PDNB, palmar/plantar digital nerve block; ASNB, abaxial sesamoid nerve block; HA, hyaluronic acid*.

All horses presented for a lameness examination within 2 weeks of onset of lameness. The severity of lameness varied from AAEP grade two to four, with a median grade of three (see Table 1). Lameness location to the distal phalanx was confirmed by anesthesia of the distal interphalangeal joint in 13 cases (all forelimbs), a palmar digital nerve block in four cases, a plantar digital nerve block in one hindlimb and an abaxial sesamoid nerve block (all hindlimbs) in four cases. In cases with a positive palmar/plantar digital nerve block, intra-articular anesthesia of the distal interphalangeal joint was not performed and, in case of a positive abaxial sesamoid nerve block, neither a plantar digital nerve block nor a distal interphalangeal joint block were performed. All horses with a positive abaxial sesamoid nerve block had a negative metatarsophalangeal joint anesthesia to ensure that the lameness was not associated with the metatarsophalangeal joint.

Radiographs were obtained with a computed radiographic system (eleven cases, Agfa DX-G) or a direct digital radiographic system (eleven cases, Tru-DR Sound). Lateromedial projections and dorsoproximal-palmaro-/plantarodistal oblique (“upright pedal”) or dorso 65° proximal-palmaro-/plantarodistal oblique projections (exposure settings 65 kV /10 mAs) were available in all cases. Weight bearing dorsopalmar/-plantar projections (exposure settings 70 kV/10 mAs) were available in 17 cases, dorsomedial-palmaro (-plantaro) lateral oblique and dorsolateral-palmaro(-plantaro) medial oblique projections (70 kV/10 mAs) were available in 13 cases as were palmaro-45°/plantaro45° proximal-palmaro-/plantarodistal oblique projections (exposure settings 50 kV/20 mAs). The projection most beneficial to the authors for the identification of an OCLL was the dorsoproximal-palmaro-/plantarodistal oblique (“upright pedal”) or dorso 65° proximal-palmaro-/plantarodistal oblique projection.

**Figure 1:**
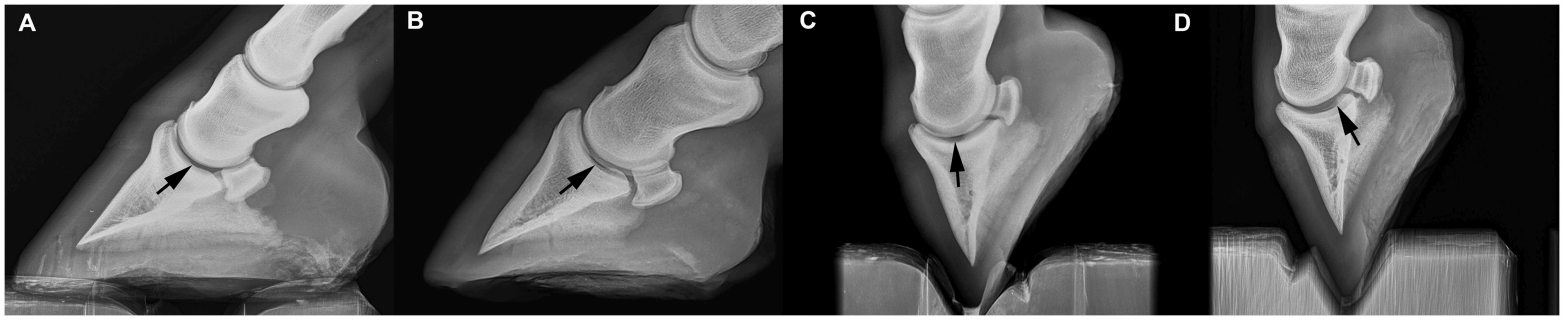
Lateromedial radiograph of the foot of four horses. **(A)** Nine year old Thoroughbred (case number 6), the articular surface of the distal phalanx shows a faint indentation. **(B)** Thoroughbred yearling (case number 18), the irregularity of the articular margin is more pronounced. **(C)** Two year old Thoroughbred (case number 22), a large defect in the articular surface is seen at the middle aspect of the distal phalanx. **(D)** Thoroughbred foal (case number 4), the defect is located in the plantar aspect of the joint.

**Figure 2:**
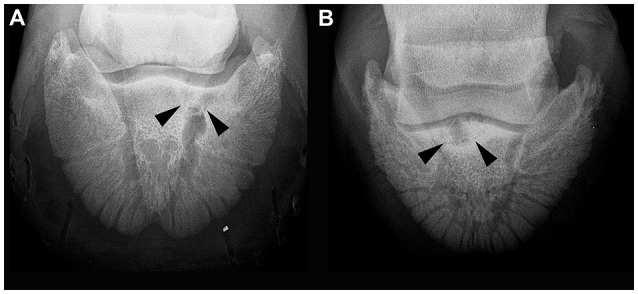
Dorso 65° proximal-palmarodistal oblique (“upright pedal”) radiographic projection of the foot of two horses. **(A)** Five year old Thoroughbred (case number 5), the osseous cyst-like lesion medial to the sagittal plane is surrounded by a faint sclerotic rim. **(B)** Thoroughbred yearling (case number 9), marked sclerosis is present in the distal phalanx surrounding the osseous cyst-like lesion.

Radiographic evaluation of the location of the OCLL within the distal phalanx were as follows: A sagittal location in 16 cases (73%), a location lateral to the sagittal plane in two cases (9%) and medial to the sagittal plane in four cases (18%). In eight cases, an indentation in the articular surface of the distal phalanx could be seen on lateromedial or oblique projections (36%; Figure 1). In only one out of the eight cases (12.5%), where the lesion could be identified on lateromedial or oblique projections, was the location of the OCLL in the plantar aspect of the joint (Figure 1). No OCLL was located in the dorsal aspect of the articular surface. The remaining seven OCLLs were located in the middle third of the distal interphalangeal joint surface (87.5%). A sclerotic rim was noted in 19 of the 22 cases (86%; Figure 2). The size of the OCLLs measured between 0.4 and 1.5 cm in diameter and was grouped in smaller than 1 cm (10 cases; 45%) or larger than 1cm (12 cases; 55%). Increased soft tissue opacity at the dorsal aspect of the distal interphalangeal joint was seen in two cases (9%). Enthesophyte formation at the dorsal aspect of the middle phalanx was appreciated in three cases (13.6%). Osteophyte formation affecting the distal interphalangeal joint was found in one case (4.5%).

Treatments used in the management of these OCLLs consisted of intra-articular medication with anti-inflammatory steroids in four cases, intra-articular injection of platelet rich plasma in four cases and intra-articular injection of anti-inflammatory steroids and hyaluronic acid in twelve cases (Table 1). All horses, following intra-articular medication, underwent 1 month of box rest followed by two of walking exercise. A more conservative approach was taken in two horses; these horses were confined to box rest for 2 months followed by 2 months of walking exercise without treatment.

Of the 22 horses with OCLL of racing age, eight horses did not start in a race after detection of the OCLL, whereas 13 horses raced at least once after detection of the OCLL. One case had not yet reached racing age at the time of submission. This resulted in 62% of horses with OCLL racing. One hundred and forty-one maternal siblings of racing age were identified, of which 119 raced at least once (84.4%). Horses were less likely to race after detection of lameness and the identification of the OCLL than their maternal siblings (OR = 0.30, 95%CI 0.11, 0.81, *p* = 0.03). The power of statistical analysis was 67.5% indicating a low statistical power. Horses with the OCLL affecting the left forelimb were two times more likely to race than if the OCLL was found in one of the other limbs (OR = 2.33, 95%CI, 1.27, 4.27, *p* = 0.02). Neither sex, mean age at the time of detection of the OCLL, if forelimb or hindlimb was affected, lameness grade at the time of detection, nor any radiographic features such as number, size (in mm), location of the OCLL within the distal phalanx (sagittal, lateral or medial), presence of a sclerotic rim and visibility of a communication between the OCCL and the articular surface were associated with the outcome of racing at least once. Two horses with an OCLL location lateral to the sagittal plane did not race after the detection of the OCLL. There was no statistical significant association between treatment and the positive outcome of racing. The two horses having had box rest only, did not race after the detection of the OCLL.

In horses with OCLL, the highest RPR ranged from forty-one to 131 with a mean of 78.77. Fifty-three racing maternal siblings were identified. The highest RPR noted for these horses ranged from zero to 121 with a mean of 74.89. The RPR was normally distributed. The combined regression analysis showed that there was no statistical significant clustering by dam (*p* = 0.15). There was no significant difference between the mean RPR of the horses with OCLLs and their maternal siblings (*p* = 0.4). The power of the statistical analysis was 7.3%. As only one significant association was found, multivariable linear regression analysis was not performed.

## Discussion

The present study assessed the association of OCLLs in the distal phalanx and the racing performance in horses from a Thoroughbred population in the UK. Of 20,056 lameness examinations performed on Thoroughbreds by one specialized equine clinic, 22 horses with OCLLs in the distal phalanx were identified. This indicates that OCLLs in the distal phalanx is a rare cause of lameness in the UK Thoroughbred population. Some authors suggest that OCLLs in the distal phalanx occur more often in warmblood horses (25), but in general the number of individuals affected by OCLLs in the distal phalanx are low and case numbers of OCLLs in the distal phalanx in previous reports were even lower (13, 17).

The results of the present study are especially important for veterinarians attempting to offer a prognosis regarding an individual's future ability to race. It has been previously reported that in 22% of lame Thoroughbred racehorses in training, lameness was sufficient to prevent racing (1). To evaluate successful performance of horses with OCLLs in the distal phalanx, we compared Thoroughbreds with OCLLs in the distal phalanx with the performance of their maternal siblings. Despite the rather low number of affected horses, we identified a difference between lame horses with OCLLs and their maternal siblings in the ability to race. Affected horses are about three times less likely to race at least once compared to their maternal siblings. This was an expected finding, but it was surprising that the majority of horses with an OCLL in the distal phalanx (13/21) participated in at least one race. Interestingly, their racing performance was comparable to their maternal siblings, despite the diagnosis of an OCLL in the distal phalanx. However, results should be interpreted carefully due to the low numbers of cases resulting in a low statistical power.

Different treatment options have been discussed in the literature. One of them is arthroscopic debridement of the OCLL. This surgical treatment option resulted in a 91% success rate of the horses returning to racing after surgery (17). Taking only the Thoroughbred racing horses in to account, seven of the nine race horses with an OCLL in the distal phalanx were successfully performing, indicating a higher success rate compared to the population included in the present study (17). Despite the promising results of surgery, a surgical treatment may not always be an option available or chosen by the owner. Financial implications and risks associated with general anesthesia and the surgical procedure may be decision-making factors. The various treatments performed and the low numbers are limitations of this study. However, of the eight cases, which did not race, two had box rest only. In this study, horses, which did not undergo intra-articular medication, did not perform.

OCLLs in the distal phalanx and also other locations may not always be associated with lameness in horses (3). The lameness associated with OCLLs is thought to be the result of synovitis and increased intra-cystic or intra-osseous pressures leading to bone marrow oedema and pain, (26, 27). It is unclear why some OCLLs are asymptomatic (11) and in the present study, no association between the grade of lameness and the outcome of racing at least once after detection of the OCLL was found. Of all horses undergoing a lameness examination, we identified only two horses with clinically silent OCLLs, which were located in the contralateral non-lame limb. The reason for this might be that only lame horses were examined radiographically and radiographs were taken according to the lameness location and did not always include the non-lame limbs. Radiography of the foot is not included in the standard pre-purchase examination protocol of juvenile thoroughbreds in the UK, as lameness and poor performance associated with the foot does not appear to play a primary role in the Thoroughbred race horse (28). The true prevalence of OCLLs in the distal limb is therefore unknown.

The diagnosis of OCLL in the distal phalanx was made based on the identification of the OCLL on radiographs. Radiographs are known to have a limited sensitivity concerning radiolucent lesions and bone mineral density must change by approximately 30–50% before osseous lesions become radiographically apparent (29, 30), therefore small or early OCLLs may remain undetectable and contribute to the number of unidentified OCLLs in the distal phalanx using radiography (19, 29). CT, low field and high field MRI are reported to be more sensitive to detect OCLLs in the distal phalanx (19, 31). Cross-sectional imaging modalities allow in addition for better assessment of a communication between OCLL and articular surfaces, exact lesion size and location as well as evaluation of the surrounding soft tissues, which may contribute or be the cause of lameness (19, 32). Despite a thorough clinical lameness examination and diagnostic anesthesia localizing the lameness to the foot, concurrent lesions in the foot cannot be excluded, based on the present data.

Despite the large caseload of lame Thoroughbred horses, the number of horses affected with an OCLL in the distal phalanx was low and this is probably responsible for the low statistical power of some results. The only significant association found in the present population of Thoroughbred horses was that horses with OCLLs in the left forelimb were more likely to race compared to horses with an OCLL in any other limb. This result cannot be adequately explained, as in the UK, unlike in many other countries, racetracks can be clock-wise and anti-clock-wise as well as in a straight line. This association is likely the result of non-normally distributed cases and does not indicate causality. Further limitations of the present study include the heterogeneity of the population such as different age, sex, different training environment and the variety of non-surgical treatments performed. This is leading to many confounding variables, which may affect the positive outcome of racing performance.

In this study, radiographic features of the OCLL were rather similar and we did not find marked variation in OCLL diameter, sclerosis and location within the distal phalanx. Radiographically visible communication with the joint was not even associated with racing performance, but an association of communication to performance would probably be assessed better using advanced imaging modalities such as CT or MRI.

## Conclusion

The present study revealed that horses with OCLLs affecting the distal phalanx were significantly less likely to race than their maternal siblings; however, the majority of these horses (62%) raced at least one. If horses with OCLLs in the distal phalanx raced after detection of the OCLL, their performance, measured as RPR, was comparable to their maternal siblings. This is important information for veterinarians working with Thoroughbred racehorses to aid them to accurately advise trainers and horse owners when facing an equine athlete with an OCLL in the distal phalanx.

## Ethics statement

For this retrospective study, the involved horses underwent diagnostic and therapeutic procedures according to best practice veterinary care and no additional examinations or interventions were performed related to the study. As a retrospective study, the study was not covered by an animal use and care protocol. For the use of clinical data and images, owners give written consent routinely at admission at the clinic.

## Author contributions

We confirm that all the authors have fulfilled the following points: Substantial contributions to the conception or design of the work, the analysis and interpretation of data for the work. Drafting the work, revising it critically for important intellectual content. Final approval of the version to be published. Agreement to be accountable for all aspects of the work in ensuring that questions related to the accuracy or integrity of any part of the work are appropriately investigated and resolved.

### Conflict of interest statement

The authors declare that the research was conducted in the absence of any commercial or financial relationships that could be construed as a potential conflict of interest. The reviewer EC and handling Editor declared their shared affiliation.
